# Special Issue: Natural Substances against Insect Pests: Assets and Liabilities

**DOI:** 10.3390/insects12030244

**Published:** 2021-03-15

**Authors:** Barbara Conti

**Affiliations:** Department of Agriculture, Food and Environment, University of Pisa, Via del Borghetto 80, 56124 Pisa, Italy; barbara.conti@unipi.it

Many insect pests directly compete with humans for food, damaging several crops in the field and during the processing and storage. Every year, about 20% of the global production of agricultural goods is lost due to more than 20,000 species of harmful insects. Moreover, some parasites can act as vectors of viruses and microorganisms, both pathogenic to humans and other vertebrates. The control of those species is, therefore, a crucial focus for the medical, veterinary, and agro-food chain operators. Scientific evidence revealed that the massive and continuous use of synthetic pesticides results in an accumulation of residues in the environment (causing air, soil, and water pollution), and have neurotoxic, carcinogenic, teratogenic, and mutagenic effects on human and non-target animals. Furthermore, the insurgence of resistance to the more commonly used chemicals in repeatedly treated pests is thoroughly stated in a multitude of scientific publications. Despite all the drawbacks reported, insect pest control still mainly relies on synthetic insecticides. Since the 1980s, research on natural products, also known as biopesticides, has highlighted their numerous helpful effects against insect pests. To fully understand and exploit the potential of natural substances, though, more improvement in the knowledge of their vast and various bioactivities is essential. Biopesticides comprise a broad group of different materials, which includes botanical products (e.g., volatile and fixed oils, vegetal extracts, hydrolates), inert dust (e.g., diatomaceous earth, granite dust, kaolin), as well as microorganisms (entomopathogenic bacteria, fungi). Because of their natural origin, biopesticides are supposed to have fewer side effects than synthetic pesticides, although they are not free from intrinsic limits. This “Natural Substances against Insect Pests: Assets and Liabilities” Special Issue (SI) addresses current basic and applied research on the isolation, chemical characterization, biochemistry, bioactivity, mode of action, benefits, and risks related to the use of natural substances as insecticides.

In detail, this SI includes studies on the bioactivities of some essential oils, such as *Origanum vulgare* [1], *Citrus reticulata*, *Melaleuca alternifolia* [2], *Mentha arvensis* [3], *Cymbopogon citratus*, *C. winterianus*, *Eucalyptus citriodora*, and *E. camaldulensis* [4]. These essential oils have been tested as insecticides and/or repellents against different Diptera species, from the pathogens carrying blowfly *Calliphora vomitoria* [1] and the mosquito *Aedes aegypti* [3,4] to the fruit fly *Drosophila suzukii* [2], demonstrating broad efficacy. One of the latest trends and challenges in the bioinsecticides field is the use of plant extracts obtained by the maceration of vegetal organs in a variety of organic or aqueous solvents. In this SI, the bioactivities of *Ajuga iva* [5], *Ludwigia tomentosa, L. longifolia* [6], *Sophora alopecuroides* [7], *Psiadia penninervia, Salvia officinalis, Ochradenus baccatus, Pulicaria crispa*, and *Euryops arabicus* [8] extracts have been evaluated. The target pests were two harmful Lepidoptera, the cotton leafworm *Spodoptera littoralis* [5] and the diamondback moth *Plutella xylostella* [6], the mosquito *Aedes albopictus* [7], the bean aphid *Aphis craccivora*, and its predator, the Neuroptera *Chrysoperla carnea* [8]. Vegetal extracts can act as insecticides, antifeedants, and, in some cases, as ecdysteroid. This latter mode of action interferes with the morphological and physiological transformations of the offspring while performing a certain selectivity towards entomophagous insects [8]. The main components of the extracts (e.g., alkaloids, flavonoids, saponins, tannins), not just the whole mixtures, can show a strong insecticidal effect, mostly depending on the route of administration. The production of natural-based products in nanoemulsion formulations, thanks to innovative nanotechnologies, could solve the miscibility and stability problems of the apolar compounds. This goal becomes particularly useful when related to mosquito larvae control in watery breeding sites [9]. The use of dusts, such as granite rock [10] and sulphur dust [11], against herbivorous pests could be exploited on some horticultural and ornamental crops and in vineyards within the integrated pest management context.

The SI also comprises two reviews: one on the bioactivity of vegetal extracts of *Tephrosia* species against stored product pests [12], and one regarding allelochemicals, the secondary metabolites produced by plants which are promising for crop protection against numerous harmful insects, thanks to their toxicity [13].

Last but not least, the SI contains a meta-analysis concerning 74 years of scientific literature, surveying over 2500 papers on botanical insecticides published between 1945 and 2019. This paper gives us a 360° view of the research in the bioinsecticides field, without neglecting the effects on non-target species, sub-lethal effects, and knowledge gaps [14].

In conclusion, this “Natural Substances against Insect Pests: Assets and Liabilities” Special Issue offers innovative empirical results and starting points to unravel the bioinsecticides’ complex bioactivity and role in the ecosystem.

I am very grateful to all the contributing authors, all the reviewers involved, and the editorial staff for their invaluable help in the assembly and editing of this very Special Issue.
